# Expression of degenerative markers in intervertebral discs of young and elderly asymptomatic individuals

**DOI:** 10.1371/journal.pone.0228155

**Published:** 2020-01-27

**Authors:** Josemberg S. Baptista, Vincent C. Traynelis, Edson A. Liberti, Ricardo B. V. Fontes

**Affiliations:** 1 Department of Morphology, Universidade Federal do Espirito Santo, Vitoria, Brazil; 2 Department of Anatomy, Instituto de Ciencias Biomedicas, Universidade de Sao Paulo, São Paulo, Brazil; 3 Department of Neurosurgery, Rush University Medical Center, Chicago, Illinois, United States of America; National Centre for Scientific Research-Demokritos, GREECE

## Abstract

Intervertebral disc (IVD) degeneration is a remodeling process mediated by several growth factors and cytokines. This process has been extensively studied in vitro and with pathologic specimens obtained during surgery for scoliosis or back pain. However, the occurrence and temporal evolution of these molecules during normal aging, particularly in the cervical segment, is not known. Our objective was to study and compare the presence of putative mediators in the IVD of young (<35 years, G1) and elderly (>65 years, G2) presumably asymptomatic individuals. Thirty C4-5 and C5-6 discs and thirty L4-5 and L5-S1 discs per group were collected during the autopsy of individuals whose family members denied a history of neck or back pain. Discs were divided into anterior, central (lumbar only) and posterior sectors for analysis. Immunohistochemistry for TNF-α, IL-1β, VEGF, NGF-β, BDNF, TIMP-1, MMP-1, -2 and -3 was performed and reactivity compared between groups and sectors. All of these molecules were detected in every disc sector of both G1 and G2. Most statistical comparisons (25/45, 55.6%) revealed an increase in mediator expression in G2 in relation to G1. Regional differences in the expression of remodeling enzymes were rare; NGF-β and BDNF had slightly higher expression in the cervical segment of elderly individuals. A senescent profile with elevated VEGF, MMP-2 and MMP-3 was observed across most G2 disc regions and were generally elevated from G1. In conclusion, the mere presence of any of the studied molecules inside the IVD cannot be considered pathologic. Expression of remodeling enzymes and inflammatory mediators is relatively similar across different vertebral segments and disc regions leading to a common degenerated pattern, while neurotrophins have slightly higher expression in cervical discs. These findings support the concept that disc remodeling in different segments follows a similar pathway that can be potentially mediated to avoid structural failure.

## Introduction

Intervertebral discs (IVDs) undergo extensive modifications during adult life, a process broadly named disc degeneration. It differs from degenerative disc disease simply by the absence of painful symptoms; there is no single molecular or structural marker to reliably differentiate one from the other [1].

Several studies have attempted to identify the mediator molecules involved in the degenerative process. Brain-derived neurotrophic factor (BDNF) and Nerve Growth Factor-Beta (NGF-β) have been identified as important neurotrophins more likely to be present in the discs of symptomatic individuals and associated with nerve ingrowth into the IVD [2,3]. Vascular endothelial growth factor (VEGF) was associated with neovascularization in herniated disc fragments and shown to modulate the actions of enzymes from the *matrix metalloproteinase* (MMP) family [4]. MMPs are putative mediators in IVD matrix turnover through degradation of proteins from both the proteoglycan and collagen families; several members of this family (especially MMPs-1, -2, -3, -7, -8 and -9) and some of their inhibitors called *tissue inhibitors of matrix metalloproteinases* (TIMPs) are overexpressed in symptomatic IVDs [5–8]. Proinflammatory cytokines such as Interleukin Beta (IL-β) and Tumor Necrosis Factor alpha (TNF-α) have been shown to play a significant role in the inflammatory process following disc herniation [9,10]. *In vitro* studies have shown that both IL-1β and TNF-α can stimulate the synthesis of BDNF, NGF-β and VEGF and regulate the expression of molecules from the MMP family [9–12].

Despite all the data generated *in vitro* and in pathological specimens, very little literature exists concerning the expression and role of these molecules during normal aging. Since prospectively recruiting asymptomatic individuals and following them until death is not feasible, a number of inadequate surrogates have been utilized for “normal” discs, such as discs obtained during surgery for scoliosis or trauma or even specimens from symptomatic individuals [10,13]. Thus we propose a model to study the expression of these mediator molecules in the IVDs of individuals from unselected autopsies whose family members were unaware of any prior history of back pain–“presumably asymptomatic” individuals. Our hypothesis is that expression of these molecules occurs during asymptomatic aging and therefore their presence in the IVD is not inherently pathologic.

## Materials and methods

This study was granted IRB approval at the Ethics Committee of Faculdade de Medicina from Universidade de Sao Paulo (CEP-FMUSP), register number 007/12. The consent was acquired through a written form with the families of the deceased. Recently-deceased (<6 hours) cadavers from unselected autopsies at the Servico de Verificacao de Obitos da Universidade de Sao Paulo were enrolled following autopsy data and a family interview were conducted to exclude any individuals with a history of neck, back, arm or leg pain, neoplasms or rheumatological conditions as previously described [14,15]. Fifteen consecutive individuals younger than 35 years of age were selected for the study and comprised Group 1 (G1). Similarly, fifteen consecutive individuals aged 65 and older were selected for Group 2 (G2). Demographic data are detailed in Table 1.

**Table 1:** Cadaver data.

	G1^a^	G2^a^	*p*^b^
Age (yrs)	31.8 +/- 2.6	78.1 +/- 7.8	<0.001
Height (cm)	172.6 +/- 8.0	166.0 +/- 9.4	0.07
Weight (kg)	72.5 +/- 14.7	68.4 +/- 22.0	0.06
Male:Female	16:4	13:7	---

^a^Average +/- standard deviation.

^b^*p*, Student’s T analysis of G1 versus G2.

*En bloc* segments of the cervical (C4-6) and lumbar spines (L4-S1) were harvested thus comprising 30 cervical and 30 lumbar discs per group. “Cervical” or “Lumbar” thus referred as the vertebral segment the disc was removed from. Specimens were assigned random identifiers and masked to researchers. G2 specimens were significantly more degenerated than G1. The accumulation of such changes was the object of an extensive multimodal analysis and are reported separately [15,16]. Briefly, morphological grading demonstrated incipient changes (Thompson 2) in 100% of G1 specimens, while advanced degenerative features (Thompson 4–5) were found in >50% of **G2** cadavers. Molecular markers relevant to the proposed pathophysiology of DDD were selected based on literature review and commercial availability of antibodies for immunohistochemistry (IHC) on human specimens. Each marker has been implicated in the degenerative process in a *remodeling* (MMP-1, -2, -3 and TIMP-1), *inflammatory* (IL-1β, TNF-α, and VEGF) or *neurotrophic* (NGF-β and BDNF) role. Five cadavers from G1 and G2 each were allocated to each marker group. Cervical discs were divided into an *anterior* and a *posterior* fragment for analysis while lumbar IVDs were divided into *anterior*, *central* and *posterior* fragments. The lumbar *central* fragment was mostly comprised of NP while all others mostly included annular material.

Immunohistochemistry (IHC) protocols were adapted to our specimens from the existing literature [7,17]. The detailed fixation, decalcification, and structural analysis protocol are described in the original paper [15]. Summarily, whole C4-6 and L4-S1 vertebral segments were fixated in 4% formaldehyde for six months, then discs and their adjacent, intact endplates were decalcified in 0.25M EDTA for 30 days and 1M EDTA for 5 days. Eight micrometer-thick semi-serial sagittal frozen sections were obtained from each disc sector and underwent IHC processing. They were sequentially submitted to the following steps, each preceded by two phosphate-buffered salines (PBS) washes using a commercially-available peroxidase-based kit (EnVision Flex, Dako, Denmark): antigen unmasking through protease digestion (0.4% pepsin in 0.01N HCl, 30 minutes), neutralization of endogenous peroxidase (1% H_2_O_2_ in PBS, 10 minutes), incubation in normal blocking serum (60 minutes), incubation with the primary (12 hours at 4 degrees centigrade–Table 2) and secondary antibodies (30 minutes, room temperature), HRP reagent (20 minutes) and the peroxidase reagent, DAB, for 1 minute. Sections were finally dehydrated and slides mounted in the usual manner [18,19].

**Table 2:** Antibodies utilized in the study.

Antigen	Antibody data
MMP-1	Santa Cruz, sc-21731
MMP-2	Santa Cruz, sc-71595
MMP-3	Santa Cruz, sc-80202
TIMP -1	Santa Cruz, sc-80365
IL-1β	Santa Cruz, sc-130323
TNF-*α*	Santa Cruz, sc-130349
VEGF	Santa Cruz, sc-57496
NGF-β	Sigma Aldrich, N3279
BDNF	R&D System, MAB248

Ten random, non-overlapping photomicrographs were obtained at 1000x magnification of each disc sector (anterior, central or posterior as described above). Histological elements distinct from the fibrocartilaginous tissue–osseous endplate, small blood vessels and nerve fibers–were not included in the photomicrographs or the immunohistochemical analysis in order to prevent bias in the expression analysis. These sections were analyzed qualitative- and quantitatively. An area-based quantification method derived from Lehr *et al*. was utilized to measure chromogen presence in each photomicrograph [15,20]. Image-analysis software measured the stained percentage of the total photomicrograph area (ImageJ, NIH, Bethesda, MD). One hundred data points were collected per antibody, per disc sector and per age group (10 photomicrographs per disc region, 2 discs per segment, 5 cadavers per marker in each group). Two negative controls per cadaver were utilized to assess and correct for abnormal chromogen presence: a double-negative, no-antibody control (Neg-Neg) to assess background chromogen deposition and a single-negative (Neg-Ab2) control with secondary antibody only (no primary antibody) to control for non-specific secondary antibody staining. In both controls, the pertinent steps were substituted with a PBS wash. Stained area values from each series with primary antibody were compiled and analyzed against the two negative controls with one-way ANOVA to assess if staining for each individual segment and antibody was statistically different than background or non-specific staining, in which case *post-hoc* Tukey analysis was used (GraphPad Prism 6, San Diego, CA).

Comparisons between G1 and G2 values were performed with Student’s T-test and are fully demonstrated below. Distribution was normal as assessed by Comparisons between disc sectors or vertebral regions, within a given age group (for example, Anterior Cervical *versus* Anterior Lumbar), were made in the same manner and commented upon when relevant to the discussion; the full results are omitted for the sake of brevity. No adjustments were made for multiple comparisons as all molecules are part of the same pathophysiological process and priority is given to the general picture *versus* any individual comparison [21]. A significance level of .05 was utilized throughout the study.

## Results

**Fig 1:**
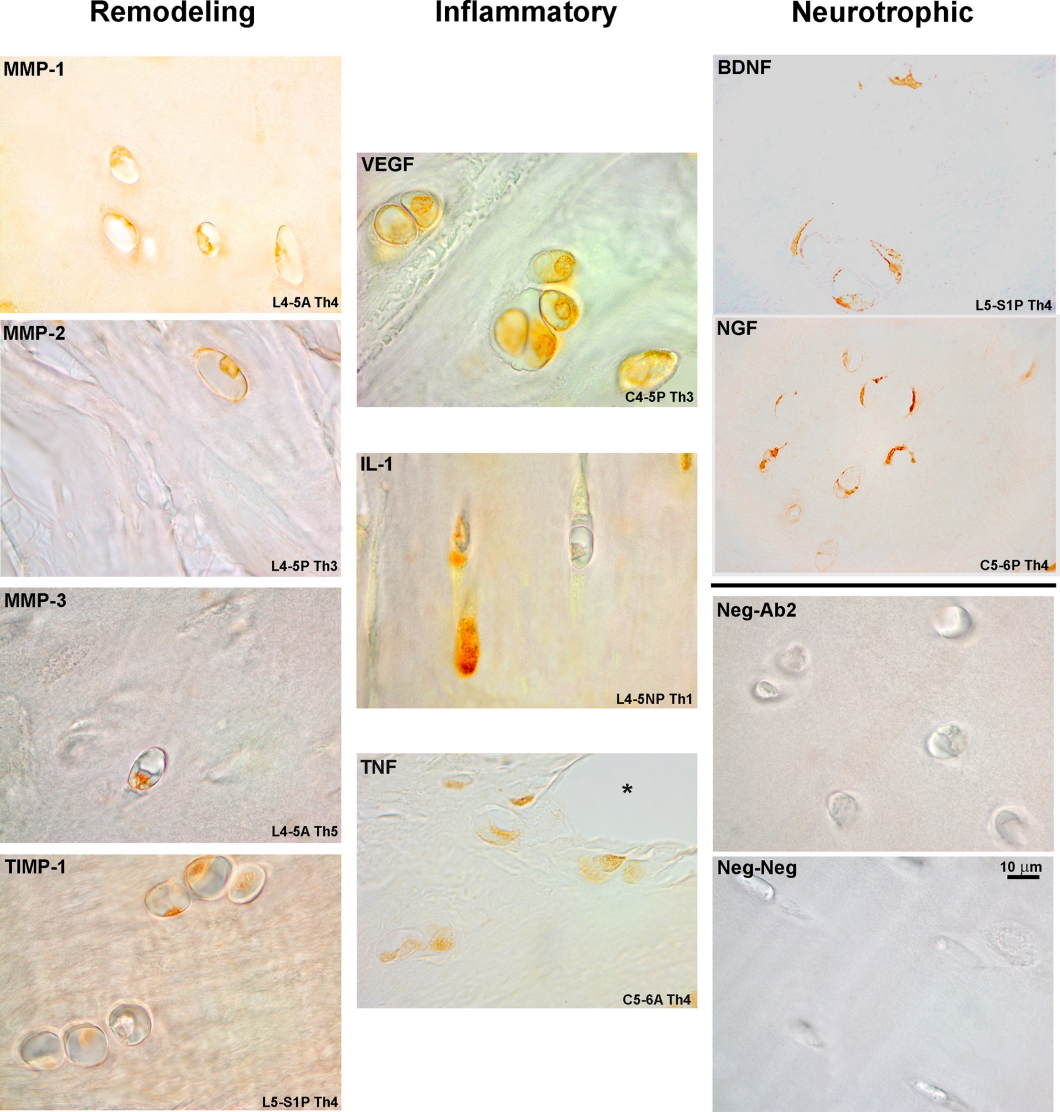
IHC staining, G1 specimens. Disc region and Thompson grade (Th) included. Reactivity was mostly intracellular but occasional ECM reactivity is shown. The asterisk denotes a fissure. Scale bar applies to all figures.

**Fig 2:**
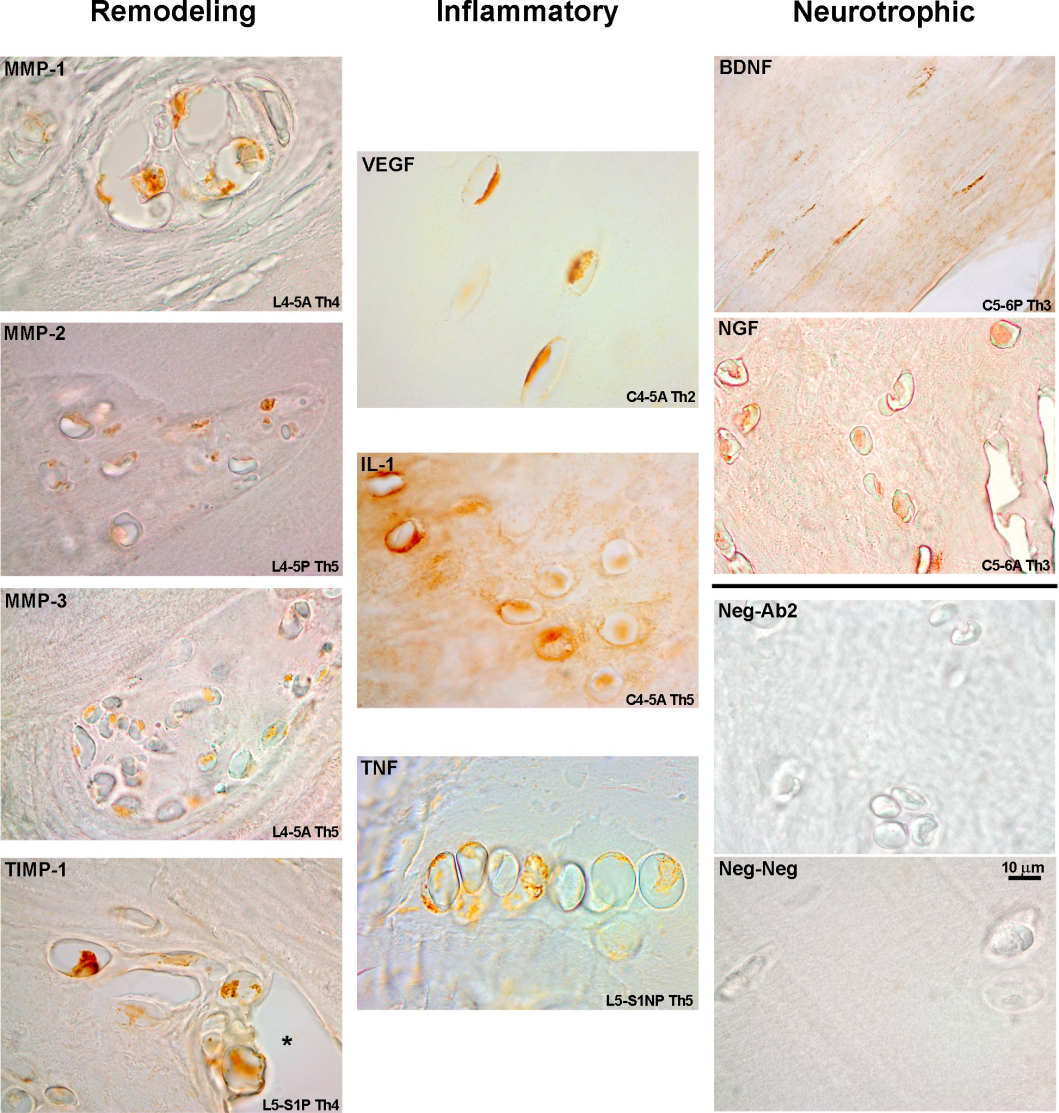
IHC staining, G2 specimens. Disc region and Thompson grade (Th) included. Reactivity is enhanced when compared to G1 specimens but the localization pattern was similar. Sporadic ECM reactivity is also found (IL-1β, BDNF). Groups of chondrocytes usually had a similar expression pattern (MMP-1, TNF-α). Scale bar applies to all figures.

Qualitative analysis of IHC staining for the mediator molecules studied here demonstrated a predominantly intracellular or membrane reactivity (Fig 1). Staining was concentrated along the more cellular regions of the IVD, especially in the vicinity of the vertebral endplate but also adjacent to fissures and remodeling areas of the IVD. Reactivity was generally congruent within groups of clustered cells in the IVD and demonstrated sparingly within the ECM (Fig 1, VEGF). Morphologically, these cells resembled chondrocytes and increased in a number closer to the vertebral endplate. G2 exhibited IHC reactivity along the same morphological lines as G1 but stained area was increased, particularly for the remodeling enzyme group (Fig 2).

**Fig 3:**
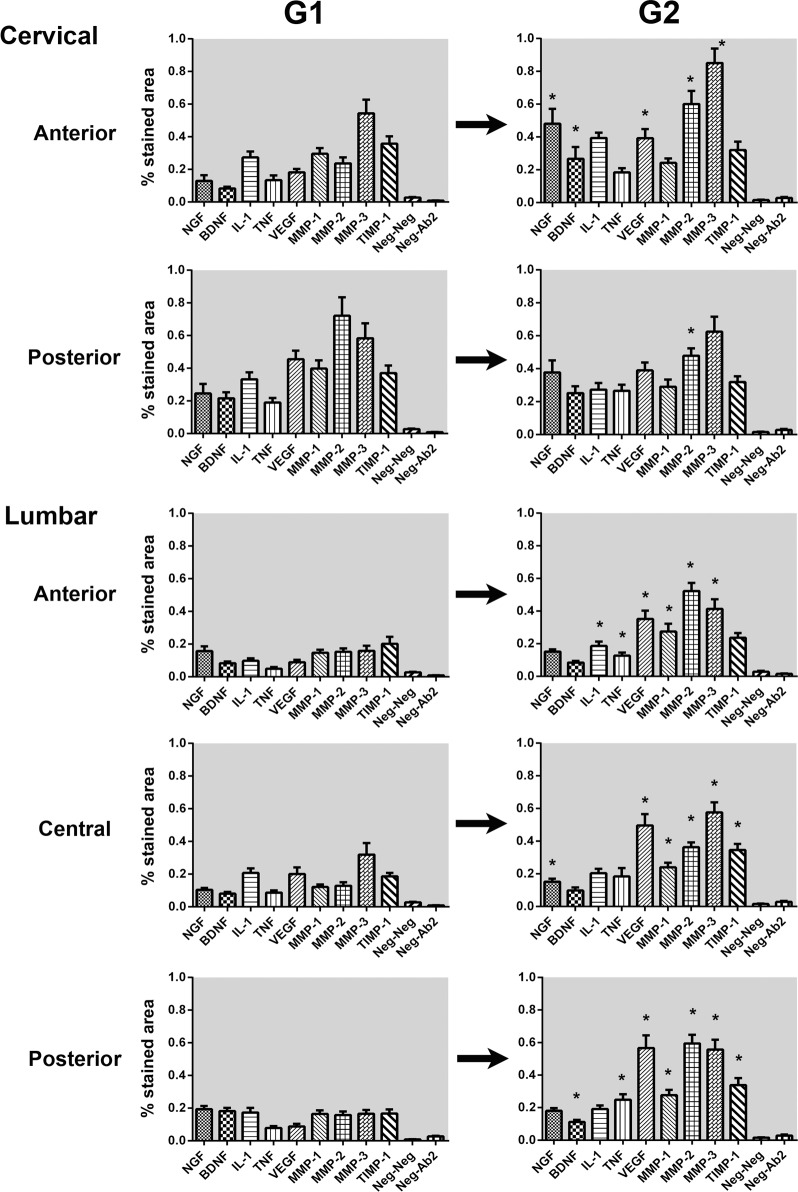
Expression of studied markers per disc region and vertebral segment. Error bars represent standard error of the mean. Asterisks mark statistical significance at .05 level for the G1 *versus* G2 comparison.

Quantitative analysis results are demonstrated in Fig 3. Comparison with negative controls demonstrated significant expression of all tested molecules in every disc region of G1 and G2. Fig 3 demonstrates how several mediators were significantly increased in G2 –out of the 45 comparisons performed between G1 and G2, 25 involved an increase in the expression of the mediator in G2, 18 were unchanged and only two had decreased levels in G2. A fairly regular pattern of elevation of VEGF, MMP-2, and MMP-3 was seen in most disc regions (4 out of 5 disc regions). The expression of MMP-1 was also significantly elevated in all lumbar fragments from G2. The disc region that the presented most stability in molecular pattern was the posterior cervical disc region, where only a slight decrease in MMP-2 expression was observed in G2. The expression of IL-1β and TNF-α was also significantly increased in sectors of the G2 lumbar AF.

Comparisons between vertebral segments (same disc region, cervical versus lumbar) and disc region (same vertebral segment, anterior versus posterior) within the same age group showed relatively few statistically significant differences. The most notable of these differences was a higher expression of BDNF and NGF-β expression in the cervical specimens of G2 (*p* < .05) when compared to the lumbar fragments from the same cadavers. MMP-1 was also noted to have a higher expression in the cervical discs of G1 but this difference was not observed in G2.

## Discussion

The presence of all molecules studied here had been varyingly demonstrated in the lumbar spine of symptomatic individuals and usually interpreted as pathologic [3,8,22,23]. This first became a matter of clinical importance in the early 2000s with the initial studies associating the presence of nerve endings and expression of NGF-β and BDNF in the periphery of discs obtained in surgery for discogenic pain [2,3,24]. On the other hand, the occurrence of these molecules in asymptomatic individuals is seldom discussed. The lack of a “baseline” comparison poses a significant problem as these molecules are involved in the normal aging of other tissues [25,26]. The utilization of inadequate surrogates for a truly normal human disc–such as discs obtained in deformity surgery or from other animal species–and suboptimal methodology–qualitative or cell-counting methods in a cell-poor tissue such as the IVD–may further complicate understanding the molecular mediators of the degenerative process [2,23,23,27].

The expression of all the studied enzymes was demonstrated in every disc region and vertebral segment, in both G1 and G2, predominantly in the cytoplasm and close to the vertebral endplate and fissures. This concentration is somewhat expected as these tend to be cell-rich areas and these molecules are growth factors and putative mediators of ECM remodeling. Our findings support that the presence of these enzymes *per se* cannot be considered a pathologic finding. Weiler *et al*. demonstrated the presence of MMP-1, -2, -3 and -9 in lumbar discs of asymptomatic individuals of similar age but the temporal evolution is slightly distinct from that shown here—in their study, the expression of MMPs generally decreased between early adulthood and senescence, with the notable exception of MMP-2 [28]. Besides methodological differences that could explain the divergent results, it should be noted that in their study degeneration was worse in younger patients—when the MMP expression was compared to degeneration and not age, it was also found to increase in a congruent manner [28].

The molecular profile of G2 as shown in Fig 3 is very similar across all disc regions, with the exception of neurotrophins. NGF-β and BDNF corresponded to the only new regional difference in G2 (cervical > lumbar). A “degenerated profile” with elevated expression of VEGF, MMP-2, and MMP-3 seemed consistent across most G2 disc regions and, except for the posterior cervical area, were significantly different from G1. Zigouris *et al*. have recently demonstrated a similar increase in MMP-1 and -3 expression with increasing age in herniated lumbar IVD fragments. Besides being obviously derived from symptomatic individuals, their study differs from ours in that degeneration was more advanced in the younger, symptomatic subgroup of his study [8]. This will be almost always the case of studies with herniated material, as the herniation itself is a defining characteristic of higher morphological grade in most scales, even though at the ultrastructural level it is a different process. Bachmeier *et al*. also demonstrated upregulation of MMP-3 and TIMP-1 mRNA levels in lumbar IVDs of symptomatic patients [13]. Weiler *et al*. have recently demonstrated strong intra-individual correlation of morphological findings across different vertebral segments; our collagen and molecular results support their findings [29]. These cervical results represent important new information as it is not known if and how the degenerative process would differ in that segment with unique load and morphology. Only two studies ever analyzed cervical discs but always in isolation and involving surgical specimens: Furusawa *et al*. demonstrated the presence of MMP-3 in herniated disc fragments, while Kokubo *et al*. confirmed the presence of TNF-α, MMP-3, VEGF and NGF-β in a significant fraction of a large number of surgical specimens from cervical spondylotic myelopathy [23,27].

The resulting “degenerated” or “senescent” profile with increased VEGF, MMP-2 and -3 allows some interesting insights when correlated with structural and collagen data demonstrated in these same specimens [15]. Even though structurally the disc may have collapsed in older individuals, from the molecular standpoint it does not exhibit a “burned-out” phenotype: the increased expression of inflammatory markers and remodeling enzymes demonstrates the remodeling process is ongoing and the disc is metabolically active even when advanced degenerative features are present. These findings support the original conclusions of Coventry *et al*. made almost 70 years regarding disc degeneration—no single morphological, imaging, ultrastructural or molecular marker can, by itself, be considered pathologic as they are present in a significant fraction of asymptomatic or unselected individuals. The proposed hypothesis is thus proven with expression of the studied molecules; furthermore, this aging pattern is reasonably consistent in both the cervical and lumbar vertebral segments.

This study has several deficiencies. There is hardly a “perfect” method for optimally collecting and processing human tissue for IHC. This design (unselected individuals at autopsy) makes for a very challenging IHC procedure–the timely collection of specimens and meticulous extraction of fixating agents are essential. The possibility of the family being unaware of prior symptoms can never be excluded; unfortunately, short of prospectively enrolling a large cohort of individuals, we see no other alternative to obtain these rare specimens. A family interview has been utilized by other authors to screen symptomatic individuals and, in our case, led to a recruitment period of 18 months to complete our G2 group despite the 12,000 annual autopsies at the University of Sao Paulo [14]. There is an inherent sampling error in this type of study–firstly, one must trust the examiner that analysis was performed diligently and incorporated relevant areas. Secondly, there is considerable leeway as to what include in the analysis since the sample (the disc) is a large, irregular structure containing multiple cell types, unlike an *in vitro* study. While nerves, blood vessels and osseous endplate can be excluded, there is no clear distinction between the cartilaginous endplate and the rest of the disc. In fact, the cartilaginous endplate can make up most of the disc in the elderly and since there is no clear distinction between disc and endplate, it can be partially captured in our analysis [30]. Our study thus provides an overall “average” expression pattern that does not capture regional variations within the disc due to more cellular areas demonstrating higher expression of markers. Finally, when designing this study, we built it with the aim of capturing differences across disc regions and vertebral segments–which explains the several disc sectors studied and a relatively low number of disc specimens per antibody class. This was necessary to capture eventual differences in marker expression according to load-bearing status and anatomy and given a limited amount of resources. Our results, however, demonstrated that “young” and “senescent” profiles are molecularly similar across different disc regions. We thus recommend that future studies focus on new markers and a relevant number of specimens but not necessarily test different disc segments or vertebral regions. With all these limitations, however, this study still amounted to more than 1000 IHC slides and the most comprehensive study of the expression of these markers in unselected autopsies to date.

While studies with asymptomatic individuals cannot *per se* identify a pathologic marker, they may still provide potential targets for therapeutic intervention by identifying key mediators of this process. Modulation of inflammatory mediators is a clinical reality: TNF-α antagonists have been used for years in ankylosing spondylitis and rheumatoid arthritis with good results, including slowing of the spinal manifestations of these diseases. Inhibition of TNF-α action by monoclonal antibodies has been shown to inhibit MMP-3 production by NP cells [11,31,32]. *In vitro* modulation of IL-1β action on NP cells has also shown promising results [11]. Treatment of cervical or lumbar degenerative disc disease through discectomy and fusion has remained conceptually the same as proposed by Cloward in the 1950s. Technical improvements in the last 60 years have resulted in a variety of different approaches that have generally made surgery safer, more effective in achieving arthrodesis and available to a wider range of patients but the fundamental principle is unchanged. It is thus natural that once fusion is achieved, however, functional outcomes have remained essentially the same while still precipitating the issue of adjacent-level disease [33–35]. It is our firm belief that DDD in the future will eventually be treated not by precipitating the end-result of degeneration with arthrodesis but at modifying the process through action on growth factors and molecules such as those studied here, in order to avoid structural failure of the intervertebral disc.

## Supporting information

S1 File Stat dataset of NGF-β. (PZF)

S2 File Stat dataset of BDNF. (PZF)

S3 File Stat dataset of IL-1β. (PZF)

S4 File Stat dataset of TNF-α. (PZF)

S5 File Stat dataset of VEGF. (PZF)

S6 File Stat dataset of MMP-1. (PZF)

S7 File Stat dataset of MMP-2. (PZF)

S8 File Stat dataset of MMP-3. (PZF)

S9 File Stat dataset of TIMP-1. (PZF)
